# Effect of eight-section brocade on bone mineral density in middle age and elderly people: Protocol for a systematic review and meta-analysis of randomised controlled trials

**DOI:** 10.1097/MD.0000000000018549

**Published:** 2020-01-03

**Authors:** Tianzhao Tian, Yingfeng Cai, Jianpeng Zhou, Baoxin Liu, Liye Chen, Min Shi, Haodong Liang

**Affiliations:** Guangzhou Hospital of Traditional Chinese Medicine, Guangzhou, Guangdong Province, People's Republic of China.

**Keywords:** bone mineral density, eight section brocade, meta-analysis, protocol

## Abstract

**Background:**

Physical therapy have an important role in preventing and managing osteoporosis (OP). A number of randomized controlled studies have indicated that eight-section brocade (ESB) could increase bone mass and alleviate pain, particularly in older women. However, there is no systematic review evaluating safety and efficacy of ESB.

**Methods:**

Relevant studies involving eight-section brocade in middle-aged and elderly individuals with osteoporosis were systematically identified from electronic databases, including EMBASE, PubMed, the Cochrane Library Chinese National Knowledge Infrastructure, Chinese Science and Technology Periodicals Database, Chinese BioMedical Database, and Wanfang Data. Inclusion criteria are randomised controlled trials of eight-section brocade that examine function and bone metabolism in middle-aged and elderly individuals with OP. The primary outcome measures will be bone mineral density (BMD), balance capacity, pain score, and adverse event including fracture during exercise. Review Manager (Revman Version 5.3) software will be used for data synthesis, sensitivity analysis, meta regression, subgroup analysis, and risk of bias assessment. A funnel plot will be developed to evaluate reporting bias and Begg and Egger tests will be used to assess funnel plot symmetries. We will use the Grading of Recommendations Assessment, Development and Evaluation system to assess the quality of evidence.

**Results:**

This paper will systematically review the existing evidence, assessing the safety and effect of eight-section brocade in middle-aged and elderly individuals with OP.

**Conclusion:**

The results of this review may help to establish a better approach to prevention of osteoporosis and osteoporotic fractures in high-risk groups and to provide reliableevidence for its further application.

**Ethics and dissemination:**

Our aim is to publish this systematic review in a peer-reviewed journal. Our findings will provide information about the safety of ESB exercises and their effect on BMD of middle-aged and elderly individuals. This review will not require ethical approval as there are no issues about participant privacy.

## Abstract 2

Supplemental Digital Content is available in the text

**Strength and limitations**

- This study will be the first review that systematically assess the efficacy and safety of EBS on decreasing bone loss in middle-aged and elderly individuals.
- The Cochrane Collaboration tool and The Grading of Recommendations Assessment, Development and Evaluation will be used to further evaluate study findings.
- This study will derived from all the available comparative data that include both English and Chinese language studies.
- There may be clinical heterogeneity because of variations in treatment frequency and duration and the use of additional therapies (eg, herbal medicine and Calcium Carbonate Chewable D3 tablet)

## 1 Introduction

Eight-section brocade (ESB) is a traditional and safe physical therapy based on the traditional Chinese Medicine theory of qi1. It is composed with 8 sections of different activities which were focus mainly on maintaining good health, accelerating rehabilitation process and fighting chronic diseases. Compared with other exercise therapies, ESB is easy to master in a short time and has fewer physical demands for patients, especially for those who were more elder.^[2]^ ESB exercises can help stretch limbs and muscles and regulate the breath, which improves the coordination of the body, harmonies qi and blood and contributes to physical and mental well-being.^[3]^ It is popular in middle aged patients because it is easy to learn and it can help with promoting the physical fitness and health.^[4]^ Many studies showed positive effect of ESB for muscular disorders that it can release symptoms and improved function of patients including knee osteoarthritis^[3,5]^ and neck pain.^[6]^ Some clinical trials have shown that ESB can substantially prevent bone loss and improved balance.^[1,7]^ However, there have been no relevant systematic reviews or meta-analyses on the effects and safety of ESB on preventing bone loss. Additionally, although ESB was popular in patients, there is insufficient evidence to support the widespread use of ESB individuals. Therefore, an objective examination on the efficacy and safety of ESB on decreasing bone loss is needed. In our research, we planned to conduct a systematic review and meta-analysis to evaluate the evidence from all available randomised controlled trials (RCTs) that evaluate EBS on decreasing bone loss.

## 2 Methods

### 2.1 Search strategy

**Figure 1:**
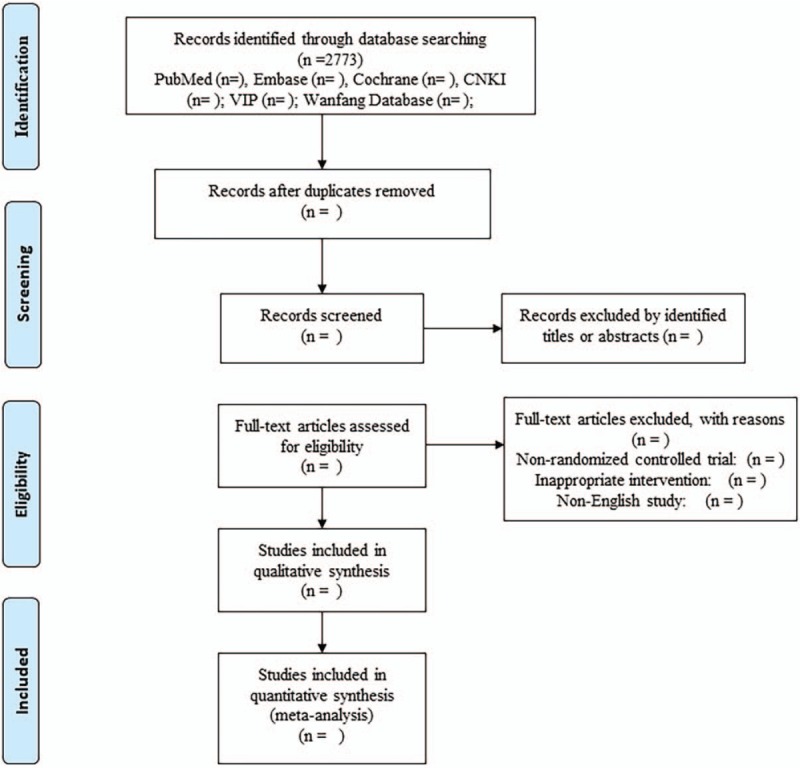
Flow diagram of the relevant study selection process.

We will conduct a systematic review and meta-analysis to identify relevant RCTs involving ESB and bone mineral density in electronic databases (Fig. 1). Two reviewers independently searched the electronic databases including Web of Science, EMBASE, PubMed, the Cochrane Controlled Trials Register, Chinese National Knowledge Infrastructure, Wanfang Data, Chinese BioMedical Database, Chinese Science and Technology Periodicals Database and the Cochrane Library up to December 2018 using the following keywords and their combinations: Baduanjin, Eight section brocade, randomized controlled trial, bone mineral density. Chinese translations of these terms will be used for the Chinese databases. The search strategy is presented in Supplemental Table 1.

### 2.2 Inclusion and exclusion criteria

The inclusion criteria will be defined before searching, and the study inclusion eligibility was determined by the following population, intervention, comparator, outcomes, and study design criteria:

1. middle-aged and elderly individuals who were able to complete or partly complete ESB.
2. RCTs that comparing ESB and other conservative treatment.
3. the age of the patients and follow-up periods were not restricted, and the publication language was not limited.

The exclusion criteria were as follows: observational studies, noncontrolled clinical trials, animal studies, and participants with histories of uterine/ovarian diseases or surgery, hormonal therapy, steroid or diuretic medication, or other diseases potentially affecting BMD and those doing regular exercise before the study.

### 2.3 Data extraction and quality assessment

Two investigators will independently extract the relevant data from each study, which included the first author's name, year of publication, country, study design, details of the intervention and control, and the follow-up duration, and outcome measurements for each study. Any uncertainty will be discussed by 2 reviewers and resolved by consensus with discussion with another reviewer. We will contact the corresponding authors of the included RCTs to obtain any missing data when necessary. The Cochrane Collaboration tool^[8]^ will be used to assess the methodological quality and risk of bias of the included studies, including randomisation, allocation concealment, blinding method, selective reporting, group similarity at baseline, incomplete outcome data, compliance, timing of outcome assessments, and intention-to-treat analysis. The Grading of Recommendations Assessment, Development and Evaluation^[9]^ approach will be used to evaluate the quality of evidence of the included studies. Reviewers will take into account limitations of the study, inconsistencies, indirect evidence, inaccuracies, and publication bias.

### 2.4 Outcome measures

The primary outcome measures that will be evaluated in our meta-analysis included BMD changes in femoral or vertebral to assess efficacy of ESB.by evaluating whether it can decrease bone loss which was measured by dual energy X-ray absorptiometry (DEXA) after treatment; and adverse events including exercise-related fracture, cardiovascular events and serious injuries to evaluate safety of ESB. The secondary outcomes will be as follows: pain Visual Analogue Scale Scores, 3-feet Up and Go Test (3UG), and one-leg Stance (OLS) to evaluate functional outcomes

### 2.5 Statistical analysis and data synthesis

The meta-analyses will be performed using Review Manager (Revman Version 5.3., the Cochrane Collaboration, Oxford, UK). Given the characteristics of the data extracted for the review, continuous outcomes will be expressed as the mean difference (MD) with 95% confidence intervals (CIs). An assumption that the standard deviations (SDs) of outcome measurements are the same in both groups will be required in all cases, and the standard deviation would then be used for both intervention groups. Heterogeneity will be assessed using the *I*^2^ statistic. *I*^2^ ≥ 50% represented high heterogeneity. To detect the impact of each data set on the overall effects of the analyses, sensitivity analysis will be performed by sequentially deleting a single study involved in the meta-analysis. Subgroup analysis will be performed based on the different follow-up periods. Risk ratios (RRs) with a 95% CI were used to assess dichotomous outcomes. The inverse variance and Mantel-Haenszel methods will be used to combine separate statistics. We will evaluate whether asymmetry was due to publication bias or to a relationship between the trial size and effect size using funnel plots. A *P* value <.05 will be considered statistically significant.

### 2.6 Patient and public involvement

No patients will be involved in this study.

## 3 Discussion

Eight section brocade has been widely practiced for more than 1000 years in China. As a type of Qigong, it is safe and easy to master, which was used for the treatment of the disease, rehabilitation, and sports medicine. EBS comprises slow, relaxing and systematic movements that are suitable for physically weak and elderly. And at the same time, it can strengthen the body muscles. Some studies showed that ESB may stimulate the immune system and enhance the power of osteogenesis by the perpendicular stress resulting from the body gravity.^[7]^ Osteoporosis (OP) and related-fracture are global public health problems. Along with increasing with the aging of population, the number of patients with OP has increased worldwide. Women aged over 60 years was estimated that they will suffer at least an osteoporotic fracture such as vertebral fracture and hip fracture in their lifetime.^[10]^ A suitable lifestyle, combination of exercise training and calcium-vitamin D play an important role in the prevention and management of osteoporosis by increasing bone mineral density and improving capacity of balance.^[11–13]^

Previous studies have showed positive effects of ESB on maintaining BMD^[1,7]^ and improving functional outcome. Therefore, it can be used to decreasing bone loss and the rate of osteoporotic fracture of patients. However, the efficacy and safety of this exercise compared with other treatment remains unknown. In addition, there is insufficient evidence that it can release pain and improved clinical outcomes. The purpose of this review is to systematically assess the effect of ESB exercises on BMD in middle aged and elderly individuals. We aim to use enough studies to ensure adequate power for the meta-analysis. We expect to find that ESB exercises can safely and effectively maintaining bone mineral and improved clinical outcome. This study will be the first review that systematically assess the efficacy and safety of EBS on decreasing bone loss in middle-aged and elderly individuals. The results of this review may help to establish a better approach to prevention of osteoporosis and osteoporotic fractures in high-risk groups and to provide reliable evidence for its further application.

## Acknowledgments

We thank American Journal Experts for its linguistic assistance during the preparation of this manuscript.

## Author contributions

**Investigation:** Min Shi.

**Methodology:** Baoxin Liu.

**Software:** Yingfeng Cai, Jianpeng Zhou.

**Visualization:** Liye Chen.

**Writing – original draft:** Tianzhao Tian.

## Supplementary Material

Supplemental Digital Content
