# Supplementary material for: Effect of eight-section brocade on bone mineral density in middle age and elderly people: Protocol for a systematic review and meta-analysis of randomised controlled trials
Source: Medicine (Baltimore). 2020 Jan 3;99(1):e18549. doi: 10.1097/MD.0000000000018549 (PMC6946291; doi:10.1097/MD.0000000000018549)
Supplement: Supplemental Digital Content [file medi-99-e18549-s001.doc]

**Table 1**

**Search Strategy for PubMed.**

| Search strategy | Search Terms |
| --- | --- |
| #1 | Eight section brocade (all fields) |
| #2 | Baduanjin (all fields) |
| #3 | Badunajin exercise (all fields) |
| #4 | Qigong (all fields) |
| #5 | simple Chinese health Qigong (all fields) |
| #6 | traditional Chinese health-promoting exercise (all fields) |
| #7 | #1 OR #2 OR #3 OR #4 OR#5 OR #6 |
| #8 | Bone mineral density (MeSH terms) |
| #9 | Bone mineral content (all fields) |
| #10 | Bone density (all fields) |
| #11 | Bone mineral density (all fields) |
| #12 | #8 OR #9 OR #10 OR#11 |
| #13 | randomised control trial (all fields) |
| #14 | RCT (all fields) |
| #15 | #13 OR #14 |
| #16 | #7 AND #12 AND #15 |
